# Neoadjuvant Bevacizumab plus Chemotherapy versus Chemotherapy Alone to Treat Non-Metastatic Breast Cancer: A Meta-Analysis of Randomised Controlled Trials

**DOI:** 10.1371/journal.pone.0145442

**Published:** 2015-12-30

**Authors:** Li Cao, Guang-yu Yao, Min-feng Liu, Lu-jia Chen, Xiao-lei Hu, Chang-sheng Ye

**Affiliations:** Breast Center, Nanfang Hospital, Southern Medical University, Guangzhou, Guangdong Province, China; Instituti Ospitalieri di Cremona, ITALY

## Abstract

**Purpose:**

Results from previous randomised controlled trials (RCTs) investigating whether the addition of bevacizumab to neoadjuvant chemotherapy (NAC) could statistically significantly increase the pathological complete response (pCR) and to identify which subgroup would benefit most from such regimens have produced conflicting results. This meta-analysis was designed to assess the efficacy and safety of bevacizumab plus chemotherapy compared with chemotherapy alone in the neoadjuvant setting.

**Methods:**

A literature search of MEDLINE, EMBASE, Web of Science, and the Cochrane library was performed to identify eligible studies. The primary endpoint of interest was pCR. The secondary endpoints were clinical complete rate (cCR), surgery rate, breast-conserving surgery (BCS) rate, and toxicity. The meta-analysis was performed using Review Manager software version 5.3.

**Results:**

Nine RCTs matched the selection criteria, yielding a total of 4967 patients (bevacizumab plus chemotherapy: 50.1%, chemotherapy alone: 49.9%). The results of this meta-analysis demonstrated that the addition of bevacizumab to NAC significantly increased the pCR rate (odds ratio [OR] = 1.34 [1.18–1.54]; P < 0.0001) compared with chemotherapy alone. Subgroup analysis showed that the effect of bevacizumab was more pronounced in patients with HER2-negative cancer (OR = 1.34 [1.17–1.54]; P < 0.0001) compared with HER2-positive cancer (OR = 1.69 [0.90–3.20]; P = 0.11). Similarly, in patients with HER2-negative cancer, the effect of bevacizumab was also more pronounced in patients with HR-negative cancer (OR = 1.38 [1.09–1.74]; P = 0.007) compared with HR-positive cancer (OR = 1.36 [0.78–2.35]; P = 0.27). No significant differences were observed between the groups with respect to cCR, surgery rate, or BCS rate. Additionally bevacizumab was associated with a higher incidence of neutropenia, febrile neutropenia, and hand–foot syndrome.

**Conclusions:**

Higher proportions of patients achieved pCR when bevacizumab was added to NAC compared with when they received chemotherapy alone; acceptable toxicities were also found. Subgroup analysis demonstrated that patients with histologically confirmed HER2-negative and HR-negative breast cancer benefited the most.

## Introduction

Neoadjuvant chemotherapy (NAC), known as primary or preoperative chemotherapy, has been widely used in patients with locally advanced breast cancer (LABC) and inflammatory breast cancer (IBC). NAC has also been gradually adopted in patients with operable breast cancer aiming to downsize the primary tumour to enable improved loco-regional control. Therefore, NAC could improve the rate of breast-conserving surgery (BCS) and decrease the need for complete axillary lymph-node dissection [1–3]. Other advantages of NAC include early evaluation of the sensitivity or resistance of each patient, which may enable the modification of ineffective treatment and the assessment of molecular changes in the tumour to identity future drug targets [4–7].

Response to NAC includes clinical and pathological aspects. Among the definitions of the response to NAC, pathologic complete response (pCR) has been shown to yield predicted improved long-term outcomes in several neoadjuvant studies and thus represents a potential surrogate marker of survival. These trials indicated that patients who achieve a pCR after NAC may had better overall survival (OS), disease-free survival (DFS) or event-free survival (EFS) compared with matched patients having only a partial pathological resonse (pPR) [6, 8, 9]. Even though long-term outcomes including OS and DFS are the most precise end-points for patients, it takes years’ follow-up to collect the data. Thus, pCR provides a valuable surrogate end-point for prognosis and for evaluation of NAC before the final survival events occur. Since recent years, multiple NAC regimens have emerged to help patients achieve pCR. In these regimens, bevacizumab is drawing increasing attention.

Bevacizumab (Avastin) was developed as a monoclonal antibody against vascular endothelial growth factor (VEGF), especially against VEGF-A, which is the isoform responsible for angiogenesis [10]. It is the first anti-angiogenesis regimen that consistently showed increased efficacy when used in combination with chemotherapy for the treatment of breast cancer [11]. Previous studies have indicated that bevacizumab can improve the progression-free survival (PFS) and the proportion of patients with an objective response rate (ORR) among patients with metastatic breast cancer (MBC) [12–15]. As a result, there has been a great deal of interest in the role of bevacizumab in the neoadjuvant setting. Hence, several randomised controlled trials (RCTs) have been conducted to evaluate the effect of bevacizumab in breast cancer [16–24].

However, results from relevant RCTs on the effect of the addition of bevacizumab to NAC have been conflicting, especially with regard to pCR. Data on pCR have varied across trials according to the definition of pCR and the molecular subtypes defined by the hormone-receptor (HR) and human epidermal growth factor receptor 2 (HER2) status. All but two RCTs were conducted in HER2-negative patients and revealed an increased pCR. In contrast, Hurvitz et al. reported a decreased pCR. Moreover, the pCR results were controversial according to the HR status in HER2-negative breast cancer, although an increase was found in the overall population. The National Surgical Adjuvant Breast and Bowel Project (NSABP) B-40 trial indicated that the addition of bevacizumab significantly increased the rate of pCR in patients with HR-positive cancer compared with HR-negative cancer. On the other hand, the results from the GeparQuinto trial showed the opposite.

Thus, questions about whether adding bevacizumab to NAC could statistically significantly increase pCR and which subgroup would benefit the most remain unanswered. To settle the disputes arising from these trials, we conducted this meta-analysis to assess the efficacy and safety of the addition of bevacizumab to NAC for non-metastatic breast cancer. Because pCR was the primary end point of our study, we will focus on the following issues: (1) whether adding bevacizumab would increase pCR, (2) whether such an increase or decrease would be statistically significant, (3) which subgroup would benefit the most according to HER2 status, and (4) which subgroup of patients with HER2-negative cancer would benefit the most according to HR status.

## Methods

### Search strategy

Eligible articles were identified by searching MEDLINE, EMBASE, Web of Science, and the Cochrane library between January 2008 and August 2015. The following medical subject heading (MeSH) terms were used: (breast OR mammary) AND (cancer OR tumour OR tumour OR adenocarcinoma OR neoplasms) AND (neoadjuvant OR preoperative) AND (bevacizumab OR avastin) AND (randomised OR random OR RCT OR randomised controlled trial OR trial OR clinical trial). There were no limitations placed on the publication language for the search. To obtain more information that may have been missed by the above methods, we reviewed the published abstracts between January 2008 and August 2015 from meetings of ASCO and ESMO. All references of the included studies were also reviewed to identify relevant citations. Two reviewers (Li Cao and Guangyu Yao) independently assessed all potentially relevant studies. In the case of any disagreements, consensus was reached by discussion between the two reviewers or decided by senior investigators.

### Selection criteria

The following inclusion criteria were applied: (1) RCTs that compared chemotherapy with or without bevacizumab as a neoadjuvant treatment for non-metastatic breast cancer patients, including IBC or LABC; (2) full papers or conference abstracts that were published online between January 2008 and August 2015; (3) studies that provided sufficiently detailed data to assess the short-term efficacy and toxicities of adding bevacizuamab to the chemotherapy treatment. Of all the data, post-therapy pCR was most essential. There was no limitation on chemotherapy regimens.

Exclusion criteria were as follows: non-randomised, single-arm clinical trials; and studies about adjuvant chemotherapy or metastatic breast cancer patients. Studies were also excluded if they did not provide essential data, and there was no response when the authors were contacted.

### Quality assessment

The Cochrane risk-of-bias tool was used to assess the methodological quality of each study. The risk of bias in each eligible trial was independently assessed by two reviewers (Li Cao and Guangyu Yao) [25].

### Outcomes of interest

The primary endpoint of interest was the total number of patients who achieved pCR. However, different studies defined pCR differently. In most of the included trials, pCR was defined as an absence of invasive breast cancer in the breast with or without ductal carcinoma in situ (DCIS) after neoadjuvant therapy, irrespective of the node (ypT0/TisN0/+). Total pCR (tpCR) was defined as the absence of invasive breast cancer in the breast and lymph nodes (ypT0/TisN0) [26]. However, some other studies did not include residual non-invasive DCIS in the definition of pCR. In this case, pCR was defined as the absence of invasive and intraductal disease in breast and lymph nodes (ypT0N0), and tpCR was defined as the absence of invasive and intraductal disease in breast, irrespective of the nodes (ypT0N0/+). Although tpCR is the best prognostic discriminator, it would be imprecise if we excluded data based on pCR in the breast only because of a smaller sample size [27]. Thus, we conducted our analysis according to both pCR definitions and molecular subtypes.

The secondary endpoints were the clinical complete rate (cCR), surgery rate, BCS rate, and toxicity (mainly grade 3 or 4 toxicities). cCR was defined as the absence of evidence of disease in the breast on ultrasonographic examination, mammographic, or physical examination [28].

### Statistical analyses

As all of the outcomes of interest were dichotomous variables, pooled odds ratios (ORs) were calculated, representing the odds of effect or toxicity occurring in the bevacizumab group compared with the chemotherapy-alone group. The random-effect model was used for the meta-analysis of dichotomous variables. Results were reported with 95% confidence intervals (CIs), and a p value of less than 0.05 was considered statistically significant if the 95% CI did not include the value “1”.

Statistical heterogeneity was calculated using Cochrane’s Q-statistic and quantified using the I^2^ statistic to evaluate the statistical heterogeneity between trials. Significant heterogeneity was considered to be present when the associated p value was below 0.10.

All calculations were accomplished using Review Manager software version 5.3.

## Results

### Study selection

**Fig 1:**
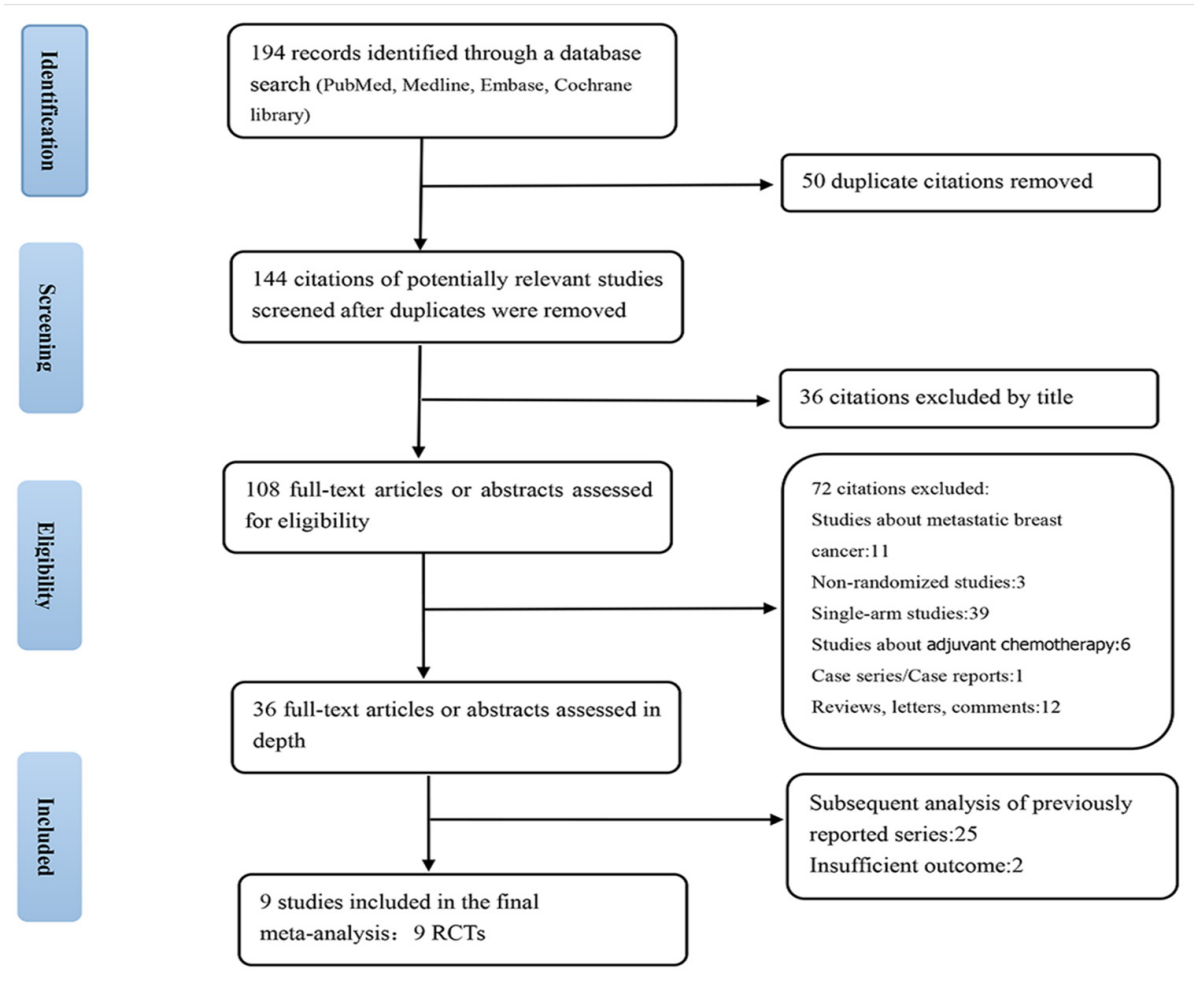
Study selection.

The electronic database searches identified 194 articles. A flow chart showing the identification of the RCTs for inclusion is depicted in Fig 1. Of these 194 articles, nine RCTs met the selection criteria and were suitable for inclusion in this meta-analysis (Table 1). Five were from peer-reviewed articles, and four were from congress abstracts. The authors' judgments with regards to the risk of bias for each included study were assessed using the Cochrane's risk-of-bias tool shown in Table 2.

**Table 1:** Baseline characteristics of the included studies.

Study	Journal	Type of Study	No. of pts (total, Exp vs Con)	pts population	Tumor stage	Treatment (Exp vs Con)	Bev schedule (cycles)	Primary end point (Exp vs Con)
**ARTemis,2015,FP**	Lancet Oncology	phase III, RCT, open label	800,399 vs 401	HER2(-) EBC	T>2cm	Bev+D-FEC vs D-FEC	15 mg/kg iv, q3w(×4)	tpCR, 22% vs 17%
**AVATAXHER, 2014,FP**	Lancet Oncology	phase II,non-comparative RCT	73,48 vs 25	HER2(+) EBC	II-III	TH+bev vs TH	15 mg/kg iv, q3w(×4)	pCR, 43.8% vs 24.0%
**CALGB 40603,2014,FP**	JCO	phase II, randomized 2×2 factorial, open label	443,222 vs 221	Operable,untreated,TNBC	II-III	wP→ddAC + Bev vs wP→ddAC; wPCb→ddAC + Bev vs wPCb→ddAC	10 mg/kg iv, q2w(×9)	pCR, 59% vs 48%
**Geparquinto,2012,FP**	NEJM	phase III, RCT	1948,974 vs 974	untreated non-metastatic HER2(-) EBC	T1-T4d	EC→T+bev vs EC→T	15 mg/kg iv, q3w(×8)	pCR, 18.4% vs 14.9%
**NSABP B-40,2012,FP**	NEJM	phase III, RCT	1206,604 vs 602	operable HER2(-)	I-III	T→AC+bev vs T→AC; TX→AC+bev vs TX→AC; TG→AC+bev vs TG→AC	15 mg/kg iv, q3w(×6)	pCR, 34.5% vs 28.2%
**S0800,2014,AB**	Cancer Research	phase II,RCT	208,96 vs 112	HER2(-) IBC/LABC	III-IV	wP→ddAC + Bev vs wP→ddAC; ddAC→wP+bev vs ddAC→wP	NR	tpCR, 38% vs 21%
**ABCSG-32,2014,AB**	Cancer Research	phase II,RCT	100,51 vs 49	HER2(+) EBC	NR	TH+bev vs TH; THN+bev vs THN	15 mg/kg	tpCR, 57% vs 49%
**Hurvitz et al., 2012,AB**	Cancer Research	RCT	58,30 vs 28	HER2(-),LABC	II-III, T>3cm	TAC+bev vs TAC	15 mg/kg (×6)	tpCR, 13% vs 19%

**No.**,number; **Pts**: patients;**Exp**, experimental arm; **Con**, control arm; **FP**, full paper; **AB**, abstract; **RCT**, randomized controlled trial; **HER2**, human epidermal growth factor receptor-2; **EBC**, early breast cancer; **TNBC**, triple-negative breast cancer; **IBC,** inflammatory breast cancer **LABC,** locally advanced breast cancer; **pCR**, pathological complete response; **tpCR**, total pathological complete response; **NR**: not reported; **Bev**, bevacizumab; **T**, docetaxel; **C**, cyclophosphomide; **Cb**, carboplatin; **E**, epirubicin; **A**, doxorubicin; **F**, fluorouracil; **G**, gemcitabine; **X**, capecitabine; **H**, trastuzumab; **P**, paclitaxel; **wP**, weekly paclitaxel; **N**, non-pegylated liposomal doxorubicin

**Table 2:** Risk of bias summary: a review of the authors' judgments with regard to the risk of bias for each item of each included trial.

Trial	Random sequence generation	Allocation concealment	Blinding of participants and personnel	Blinding of outcome assessment	Incomplete outcome data	Selective reporting
**AVATAXHER, 2014**	Low	High	High	Low	Low	Low
**ARTemis,2015**	Low	Unclear	Unclear	Unclear	Low	Low
**CALGB 40603,2015**	Low	Unclear	Unclear	Unclear	Low	Low
**Geparquinto,2012**	Low	Unclear	Unclear	Low	Low	Low
**NSABP B-40,2012**	Low	Unclear	Unclear	Unclear	Low	Low
**NeoAva,2014**	Low	Unclear	Unclear	Unclear	Low	Low
**S0800,2014**	Low	Unclear	Unclear	Unclear	Unclear	Low
**ABCSG-32,2014**	Low	Unclear	Unclear	Unclear	High	Low
**Hurvitz et al, 2012**	Low	Unclear	Unclear	Unclear	High	Low

### Characteristics of the included studies

Table 1 presents the baseline characteristics of the included studies. A total of 4967 patients were included, with 2490 in the bevacizumab arm and 2477 in the chemotherapy-alone arm. In terms of phase, five trials involved phase II, three involved phase III, and one was unknown. Only two trials focused on the HER2-positive patient population, and the other seven focused on HER2-negative patients. Details relating to HR status were available in all five trials presented in a publication but not in the abstracts. Three trials focused on operable patients only, whereas four included IBC/LABC patients, and this detail was unknown in one trial. Trials with full papers also provided details about histological type and grade. More than 70% of patients in the latter were diagnosed with invasive ductal carcinomas, and about 80% were diagnosed with histological grade 2 or 3. The majority of the NAC regimens comprised anthracyclines and taxanes. Other regimens contained carboplatin, fluorouracil, gemcitabine, capecitabine, and trastuzumab. More information is shown in Table 1.

### Outcomes of interest

**Fig 2:**
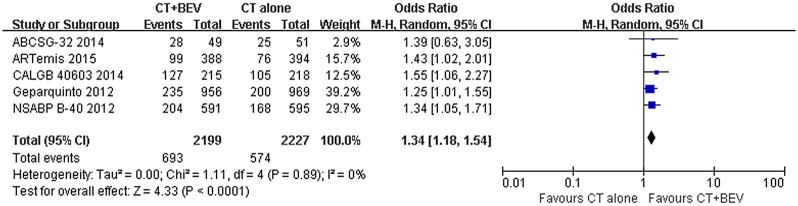
Forest plot of odds ratio on pCR in the overall population.

**Fig 3:**
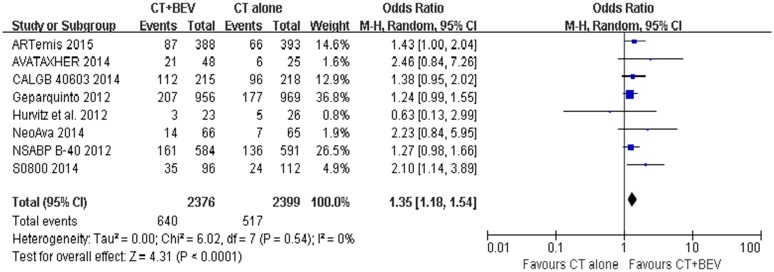
Forest plot of odds ratio on tpCR in the overall population.

**pCR. Overall population:** The incidence of pCR was reported in five of the trials. In the included trials, pCR ranged from 24.6% to 59% in the bevacizumab group and from 19% to 49% in the chemotherapy-alone group. The pooled estimate including 4426 patients evaluated for the pCR rate showed an increased pCR rate in the bevacizumab group (OR = 1.34 [1.18–1.54]; P < 0.0001) (Fig 2). Eight trials reported the tpCR rate, which ranged from 13% to 52% in the bevacizumab group and from 10.6% to 44% in the chemotherapy-alone group. Similar to the results for pCR, the pooled analysis including 4775 patients showed an increased tpCR rate in the bevacizumab group (OR = 1.35 [1.18–1.54]; P < 0.0001) (Fig 3).

**Fig 4:**
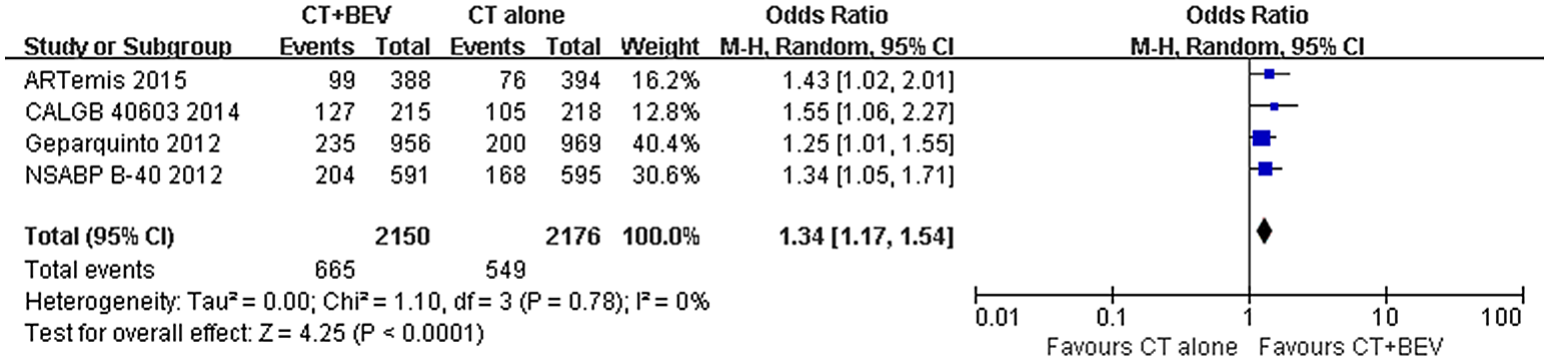
Forest plot of odds ratio on pCR in HER2-negative patients.

**Fig 5:**
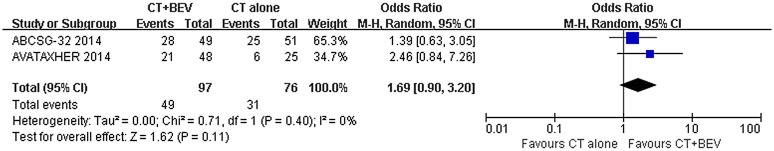
Forest plot of odds ratio on pCR/tpCR in HER2-positive patients.

**HER2 status:** A pooled subgroup analysis on the pCR according to HER2 status was also conducted. The pCR analysis of the HER2-negative patients was based on four trials. The results showed that pCR was statistically higher when bevacizumab was added to NAC (OR = 1.34 [1.17–1.54]; P < 0.0001) (Fig 4). Analysis of the tpCR based on seven trials in this subgroup yielded a similar result (OR = 1.34 [1.16–1.53]; P < 0.0001). In total, there were only two trials conducted in patients with HER2-positive breast cancer; one of them reported pCR, whereas the other reported tpCR. Thus, we were unable to conduct the analysis according to pCR definition. To analyse the HER-positive subgroup, we ignored the difference between pCR and tpCR. However, no statistically significant difference was found between the bevacizumab and the chemotherapy-alone group (OR = 1.69 [0.90–3.20]; P = 0.11) (Fig 5).

**Fig 6:**
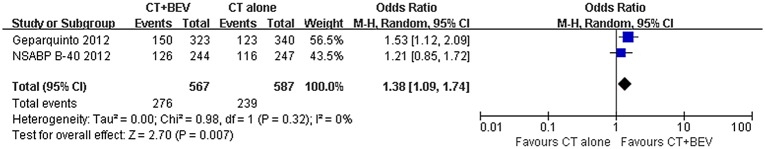
Forest plot of odds ratio on pCR in HER2-negative/HR-negative (TNBC) patients.

**Fig 7:**
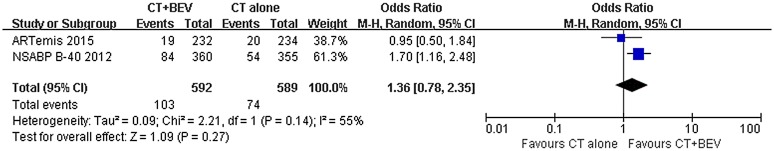
Forest plot of odds ratio on pCR in HER2-negative/HR-positive (Luminal) patients.

**HR status:** Subgroup analyses on pCR or tpCR were performed according to HR status in the HER2-negative patients. Analysis based on four trials showed that pCR in HER2-negative/HR-negative (TNBC) patients was statistically higher when bevacizumab was added to chemotherapy (OR = 1.38 [1.09–1.74]; P = 0.007) (Fig 6). As data on tpCR in TNBC patients were available only in one trial, we could not analyse the tpCR in this subgroup. On the other hand, the results in the HER2-negative/HR-positive (Luminal) patients revealed no difference in the analyses with regard to either pCR (OR = 1.36 [0.78–2.35]; P = 0.27) (Fig 7) or tpCR (OR = 1.15 [0.78–1.71]; P = 0.47). In terms of HER2-positive patients, analyses of pCR or tpCR according to the HR status were impossible because of insufficient data.

### cCR

**Fig 8:**
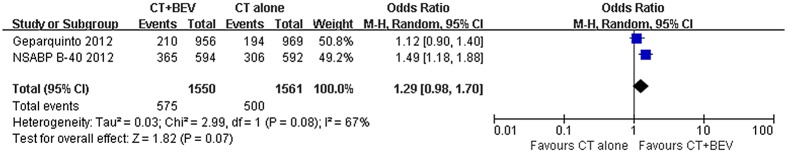
Forest plot of odds ratio on cCR.

The incidence of cCR was reported in two trials. A meta-analysis based on data from 3111 patients showed that the cCR increased by 29% when bevacizumab was added to chemotherapy, which reflected no significant difference (OR = 1.29 [0.98–1.70]; P = 0.07) (Fig 8). However, the heterogeneity between the groups was statistically significant (χ2 statistic = 2.99, P = 0.08; I2 = 67%).

### Surgery and BCS rate

**Fig 9:**
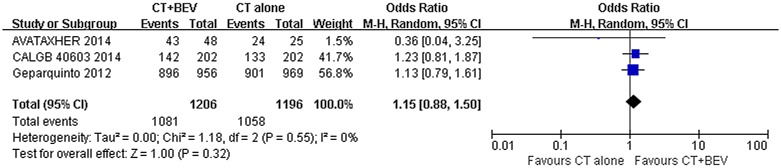
Forest plot of odds ratio on surgery rate.

**Fig 10:**
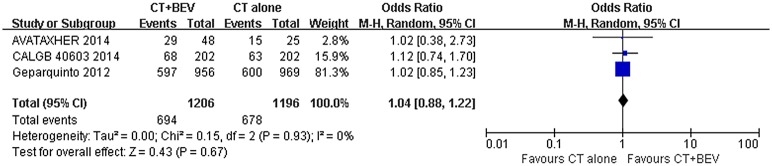
Forest plot of odds ratio on BCS rate

The analysis of surgery rate analysis was based on two trials including 1998 patients. The analysis identified a slight increase in the surgery rate, but this difference was not significant (OR = 1.15 [0.88–1.50]; P = 0.32) (Fig 9). The meta-analysis of BCS rate from 1998 patients in 2 trials also showed a slight increase by the addition of bevacizumab. Similarly, no significant difference was found (OR = 1.02 [0.85–1.23]; P = 0.81) (Fig 10). As listed in Table 3, only 4 trials provided details on surgical end points.

**Table 3:** Mastectomy and BCS rate.

Trial/Mastectomy or BCS rate (n,%)	CT alone		CT+BEV	
	Mastectomy	BCS	Mastectomy	BCS
**AVATAXHER, 2014**	9/25 (36.0%)	15/25 (60.0%)	14/48 (29.2%)	29/48 (60.4%)
**ARTemis,2015**	193/378(51.1%)	Not reported	185/386(47.9%)	Not reported
**CALGB 40603, 2015**	70/202 (34.7%)	63/202 (31.2%)	74/202 (36.6%)	68/202 (33.7%)
**Geparquinto, 2012**	301/969 (31.1%)	600/969 (61.9%)	299/956 (31.3%)	597/956 (62.4%)

### Toxicity

The incidence of grade 3 or 4 toxicities was reported in all five trials in the full papers. The results are shown in Table 4. There was no difference between the groups in terms of the incidence of anaemia (OR = 1.27 [0.71 to 2.26], P = 0.42), nausea (OR = 1.15 [0.48 to 2.78], P = 0.75), vomiting (OR = 1.86 [0.88 to 3.93], P = 0.10), diarrhoea (OR = 1.42 [0.34 to 5.87], P = 0.63), peripheral neuropathy (OR = 1.26 [0.79 to 2.03], P = 0.34), or mucositis (OR = 3.64 [0.73 to 18.22], P = 0.12). However, a meta-analysis demonstrated that bevacizumab plus chemotherapy treatment significantly increased the incidence of the following toxicities compared with chemotherapy alone: neutropenia (OR = 1.18 [1.03 to 1.36], P = 0.02), febrile neutropenia (OR = 1.99 [1.52 to 2.60], P < 0.00001), and hand–foot syndrome (OR = 1.63 [1.21 to 2.20], P = 0.001).

**Table 4:** Toxicity analysis comparing CT+BEV Versus CT alone.

Toxicity and Maximal Grade	No. of studies	No. of events	No. of patients	OR [95%CI]	p-value	HG χ^2^	HG *P*	*I* ^*2*^ *(%)*
**Hematologic**								
Neutropenia, 3–4	5	2292	4357	1.18 [1.03 to 1.36]	0.02	2.02	0.73	0
Febrile neutropenia, any	3	262	2432	1.99 [1.52 to 2.60]	<0.00001	0.19	0.09	0
Anemia, 3–4	3	50	2429	1.27 [0.71 to 2.26]	0.42	1.26	0.53	0
Thrombocytopenia, 3–4	2	124	2357	2.02 [1.00 to 4.07]	0.05	2.81	0.09	64
**Gastrointestinal**								
Nausea, 3	3	50	1291	1.15 [0.48 to 2.78]	0.75	2.20	0.14	55
Vomiting, 3–4	3	31	1291	1.86 [0.88 to 3.93]	0.10	0.16	0.69	0
Diarrhoea, 3–4	3	45	1291	1.42 [0.34 to 5.87]	0.63	2.89	0.09	65
**Others**								
Peripheral neuropathy, 3–4	3	72	2409	1.26 [0.79 to 2.03]	0.34	0.41	0.82	0
Fatigue, 3–4	3	131	1291	1.46 [1.01 to 2.11]	0.05	0.99	0.61	0
Mucositis, 3–4	2	190	2360	3.64 [0.73 to 18.22]	0.12	4.39	0.04	77
Hand–foot syndrome, 3	3	194	3180	1.63 [1.21 to 2.20]	0.001	0.11	0.95	0
Surgical complications	4	150	2435	2.38 [1.04 to 5.47]	0.04	6.97	0.07	57

## Discussion

As the previous RCTs that evaluated the efficacy of bevacizumab in breast cancer have produced conflicting results, we performed a meta-analysis to assess the efficacy and safety of bevacizumab plus chemotherapy compared with chemotherapy alone in the neoadjuvant setting. Our study found that the addition of bevacizumab can significantly increase the pCR, particularly in HER2-negative and HR-negative breast cancers, with acceptable toxicities.

From the seven trials that reported tpCR, only Hurvitz et al. reported a decreased tpCR when adding bevacizumab (9% decrease). However, our analysis showed a significantly increased tpCR rate for the addition of bevacizumab, which is in accordance with the results from the other published trials. The reason for the conflicting results might be the small simple size of the Hurvitz et al. trial. Additionally, patients in that study were treated with different doses of bevacizumab, whereas those participants were excluded from our analysis. Only patients treated with the standard dose of bevacizumab (15 mg/kg) were included in our analysis. Six trials reported pCR, and all demonstrated an increased effect. The p value was reported in four of the six trials. ARTemis, GeparQuinto, and B-40 trials showed a statistically significant improvement in pCR, whereas CALGB 40603 showed an increased pCR without a significant difference. Our analysis was the same as the former three trials, revealing a significantly increased pCR [16–20, 23].

With regard to the hypothesis that certain subgroups would benefit to a greater extent from adding bevacizumab to NAC, we conducted analyses on pCR and tpCR according to HER2 and HR status. The results demonstrated that patients with HER2-negative cancer benefited to a greater extent than those with HER2-positive cancer. Of the trials conducted in HER2-negative patients, three had sufficient data on pCR according to HR status. As discussed, GeparQuinto and B-40 were associated with conflicting results. Subgroup analysis in the NSABP B-40 trial showed that the effect of bevacizumab on pCR (ypT0Tis N0/+) was greater in patients with HR-positive cancer (15.1% vs. 23.2%, P = 0.007) compared with those with HR-negative cancer (47.1% vs. 51.5%, P = 0.34). However, in the GeparQuinto trial, the difference of pCR (ypT0N0) was greater in patients with HR-negative cancer (27.9% vs. 39.3%, P = 0.003) compared with HR-positive cancer (7.8% vs. 7.7%, P = 1.00). The ARTemis trial yielded conclusions similar to those with GeparQuinto. However, ARTemis divided participants based on oestrogen receptor (ER) status according to the Allred score, and we were unable to use the data based on patients in the ER-negative or the ER-weakly-negative subgroup [16, 18, 20].

Therefore, our analysis of the pCR in patients with HER2-negative/HR-negative tumours included only the B-40 and GeparQuinto trials. As shown, the pCR from B-40 was much lower than that from GeparQuinto. This could be the result of different definitions of pCR, which were stricter in the GeparQuinto trials. Thus, we searched the supplementary analysis of GeparQuinto (GBG 44), identifying data on pCR, which was defined as ypT0Tis N0/+ (36.2% vs. 46.4%, P = 0.009) [29]. Finally, our meta-analysis showed a significant improvement in the pCR of patients with TNBC tumour. It is regrettable that we could not analyse the tpCR in TNBC patients because only one trial contained sufficient data. Likewise, subgroup analyses were performed in patients with HER2-negative/HR-positive (Luminal) tumours. However, analyses revealed a positive trend that did not reach statistical significance for either pCR or tpCR. Thus far, we were able to conclude that the observed effect of bevacizumab on increasing the pCR was derived primarily from patients with histologically confirmed TNBC tumours. The reason for this could be the frequent activation of the genes involved in angiogenesis in TNBC [30]. There is also other evidence to suggest that ER-negative tumours tend to have a higher pCR than ER-positive tumours in response to chemotherapy [31–33]. This is of great clinical importance in that the prognosis of patients with TNBC is relatively poor, as conventional chemotherapy remains the only available systemic treatment option [34]. Recent studies confirmed the result that the pCR was higher for the TNBC subtype, further indicating that a higher pCR strongly predicted improved survival in this subtype. Nevertheless, our study is different in that the GeparQuinto (GBG 44) trial yielded a similar conclusion in the HER2-positive subtype [4, 7].

Of all 4967 patients included in this study, only 173 were confirmed with histological HER2-positive breast cancer. The relevant two trials both revealed a positive trend without reporting the p value. Unfortunately, our subgroup analysis showed that, although there was an increased pCR, the effect failed to reach statistical significance. Considering the small sample size and the lack of pCR definition, we considered the result to be less convincing. It is clear that larger, randomised trials are needed to confirm the effect of bevacizumab in this subtype. However, conflict remains because trastuzumab is the standard HER2-directed therapy, rendering the assessment of bevacizumab difficult.

As discussed, the pCR varied across trials. There may be several reasons for the differences in the pCR presented in the included trials. First, the pCR data used may have varied due to different definitions. Although our pCR analysis was divided into pCR and tpCR groups, inaccuracy remains possible. Evidence has demonstrated that a definition of pCR as ypT0N0 can best discriminate between patients with favourable and unfavourable outcomes [5, 26, 27]. As a result, we strongly suggest that this definition be used in later RCTs to make the comparison of pCR between different trials easier. Second, the included trials were conducted in different patient populations that varied in sample size, clinical stage, histological grade, and molecular subtypes. Last, but not least, the chemotherapy regimens varied in type, dose, cycle, sequential order, treatment interval and compliance.

In addition to pCR, other effects of neoadjuvant bevacizuamab plus chemotherapy include the cCR, surgery rate, and BCS rate. Surgical results were collected by reviewing full papers as well as relevant supplementary analysis [16–24, 35]. However, our meta-analysis found no evidence of any difference on these end points between the bevacizumab and chemotherapy-alone groups. As listed in Table 3, the percentage of patients who underwent BCS increased little in the experimental arm. Fortunately, researchers in CALGB 40603 found a 38% rate of conversion from BCS-ineligible to BCS-eligible with the addition of bevacizumab, compared with a 33% rate in the control arm. But compared with the other two included trials (AVATAXHER and Geparquinto), the BCS rate in CALGB 40603 was much lower. Previous neoadjuvant trials using various regimens also drew different BCS rates, ranging from 63–89% after NAC [36–38]. Although NAC is believed to be advantageous in increasing the BCS rate, this end point is influenced by multiple factors, including surgeons’ experience, the different criteria used to conduct BCS, patients’ fear of cancer recurrence, and BRCA-related genetic information [39].

To date, only one of our included trials reported data about long-term outcomes. As reported by the GeparQuinto study group, with a median follow-up of 3.8 years, 3-year DFS and 3-year OS was 80.8% and 89.7%, respectively. However, no difference was found for the addition of bevacizumab for either DFS (HR = 1.03 [0.84–1.25]; P = 0.784) or OS (HR = 0.97 [0.75–1.26]; P = 0.842) [40]. A possible explanation for the negative response on long-term survival may be the discontinuation of bevacizumab after surgery. Nevertheless, it remains to be seen whether the significant increase in pCR when adding bevacizumab to chemotherapy will lead to improved patient long-term survival benefits.

This study has shown that the addition of bevacizumab to NAC did not increase the incidence of most types of grade 3 or 4 toxicities. However, adding bevacizumab was found to increase the incidence of neutropenia, febrile neutropenia, and hand–foot syndrome, which were slight, clinically manageable, and less life-threatening. There are insufficient data from the included trials to perform a meta-analysis on cardiac toxicities, although the toxicity of bevacizumab can be fatal. However, the original data in the included trials showed that cardiac toxicities, such as arterial hypertension, congestive heart failure, and left ventricular systolic dysfunction, occurred infrequently and were acceptable. We were also unable to assess other toxicities, including bleeding, because of insufficient data.

There are several limitations to this study that need to be addressed. First, this meta-analysis is a retrospective study; thus, bias resulting from incomplete outcome data and selective reporting cannot be excluded. Second, the number of trials included was relatively small; this was particularly true of those focused on patients with HER2-positive breast cancer. Third, as the main end point of our study, the pCR varied from trial to trial due to the definition of pCR, the patient population, and the type of tumour. Owing to inadequate data, we were able to perform only subgroup analysis on molecular subtypes of breast cancer. Therefore, a meta-analysis based on sufficient data is needed to identify subgroups that could significantly benefit from the addition of bevacizumab to NAC in terms of to these factors [41–43]. To gain an improved understanding of the effect of adding bevacizumab to NAC, RCTs (NCT01190345 and NCT00203372) are still ongoing, and we suggest that more large trials to be conducted.

## Conclusion

In a neoadjuvant setting, bevacizumab plus chemotherapy compared with chemotherapy alone can help higher proportions of patients with non-metastatic breast cancer achieve a pCR, without increasing most of the grade 3 to grade 4 toxicities. The effect of bevacizumab was derived primarily from patients with histologically confirmed HER2-negative and HR-negative breast cancer.

## Supporting Information

S1 PRISMA Checklist (DOC)
